# *Staphylococcus Aureus* Osteomyelitis as an Inducer of Tolerance to *Escherichia Coli* Pyelonephritis: an Experimental Study

**DOI:** 10.1038/s41598-020-58420-w

**Published:** 2020-01-28

**Authors:** Stavros Goumenos, Olga Savvidou, George Renieris, Theologia Gkavogianni, Panayiotis J. Papagelopoulos, Evangelos J. Giamarellos-Bourboulis

**Affiliations:** 1 1st Department of Orthopedics, National and Kapodistrian University of Athens, Medical School, Athens, Greece; 2 4th Department of Internal Medicine, National and Kapodistrian University of Athens, Medical School, Athens, Greece

**Keywords:** Cytokines, Infection, Inflammation, Infectious diseases

## Abstract

The high incidence of osteomyelitis in vulnerable populations like those with multiple injuries or elderly undergoing joint arthroplasties generates the question what may be their responses to subsequent infection by high virulent isolates. Rabbits were subject to two operations at three week intervals; sham osteomyelitis and sham pyelonephritis (group S); sham osteomyelitis and *Escherichia coli* pyelonephritis (group P); and *Staphylococcus aureus* osteomyelitis and *E. coli* pyelonephritis (group OP). Survival was recorded; cytokine stimulation of circulating mononuclear cells (PBMCs) and tissue myeloperoxidase (MPO) activity and bacterial growth were monitored. In some experiments, dalbavancin treatment was given before pyelonephritis. Healthy PBMCs were pre-treated with bone homogenate, *S. aureus* or both. Mortality of groups S, P and OP after induction of pyelonephritis was 0%, 50% and 8.3% respectively. Tumour necrosis factor-alpha (TNFα) production by PBMCs was significantly lower in the OP group at 48 hours. *E. coli* bacterial load was similar in groups P and OP at death or sacrifice whereas the MPO activity of group OP was decreased. Production of TNFα was further decreased among dalbavancin treated rabbits; in these rabbits tissue MPO was increased. TNFα production decreased when healthy PBMCs pre-treated with bone homogenate, *S. aureus* (HKSA) or both were stimulated with *E. coli* (HKEC); production was further decreased in the presence of anti-TLR4 and anti-TLR9. It is concluded that staphylococcal osteomyelitis modulated the innate immune responses of the host leading to protection from death by highly virulent *E. coli*. Tolerance to TLR ligands is the most likely mechanism of action.

## Introduction

Osteomyelitis is an uncommon infection that mandates prolonged antimicrobial treatment and that often results in patient disability. Osteomyelitis usually runs in chronicity and complicates bone surgery for fractures or joint replacement^1^. As a consequence, the common profiles of patients who develop osteomyelitis are either young people with multiple trauma undergoing bone fracture fixation and who remain post-operatively in an Intensive Care Unit environment^2^ or elderly who are operated for hip or knee joint arthroplasty^3^. In these patients the immune system is in constant exposure to the antigen stimuli of *Staphylococcus aureus* that is the commonest pathogen. Due to their profile these patients are prone to subsequent severe infections like bacteremia, lung infection and urinary tract infection that may put their life in danger^4,5^. Severe infection i.e. sepsis develops as a result of the complex orchestration of host immune responses leading to the release of pro-inflammatory and anti-inflammatory cytokines. Perhaps the most critical step for the activation of the innate immune response is recognition of pathogen-associated molecular patterns (PAMPs) by pattern recognition receptors (PRRs) of blood monocytes and tissue macrophages^6^. In that case, it is questionable how recognition of PAMPs of *S. aureus* in a patient with chronic osteomyelitis may modulate immune response to a subsequent infection.

The purpose of this study was to investigate the modulation and outcomes of innate immune responses to experimental *Escherichia coli* pyelonephritis after preceding implant-related *S. aureus* osteomyelitis.

## Results

**Figure 1:**
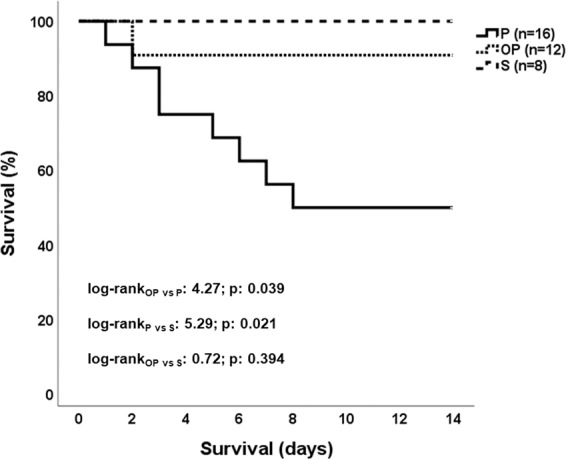
Survival of study groups Rabbits were divided into three groups: S subject to sham osteomyelitis followed three weeks later by sham pyelonephritis; P: subject to sham osteomyelitis followed three weeks later by *Escherichia coli* pyelonephritis; and OP: subject to *Staphylococcus aureus* osteomyelitis followed three weeks later by *E. coli* pyelonephritis. The log-rank tests and the p-values of the indicated comparisons are provided.

All animals survived at good condition the first operation of induction of osteomyelitis by *S. aureus* whatever allowed them to become subject to a second operation after three weeks. At that time point animals of group S already subject to sham osteomyelitis were subject to sham pyelonephritis; animals of group P already subject to sham osteomyelitis were subject to *E. coli* pyelonephritis; and animals of group OP already subject to *S. aureus* osteomyelitis were subject to *E. coli* pyelonephritis. At the end of the 14-day follow-up from the second operation mortality was 0%, 50% and 8.3% respectively (Fig. 1).

**Figure 2:**
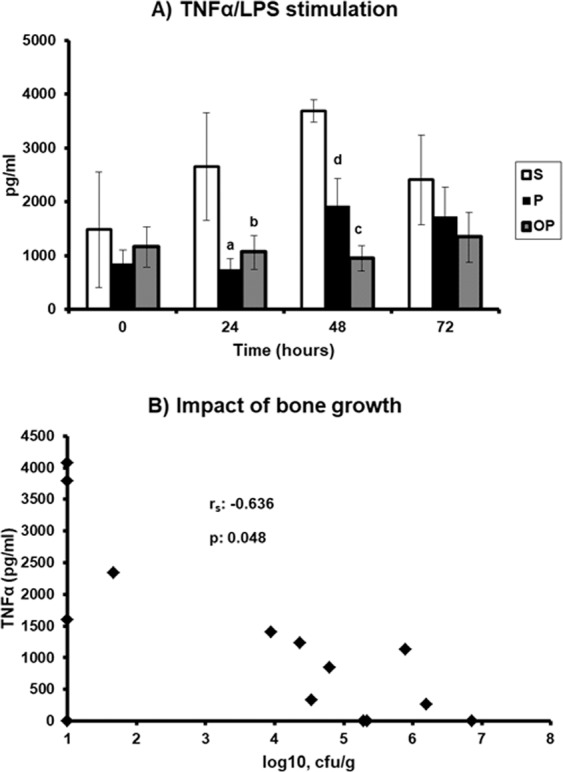
Production of tumour necrosis factor-alpha by the peripheral blood mononuclear cells (PBMCs) Rabbits were divided into three groups: S subject to sham osteomyelitis followed three weeks later by sham pyelonephritis; P: subject to sham osteomyelitis followed three weeks later by *Escherichia coli* pyelonephritis; and OP: subject to *Staphylococcus aureus* osteomyelitis followed three weeks later by *E. coli* pyelonephritis. (**A**) PBMCs were isolated at serial time points following bacterial challenge and stimulated with bacterial lipopolysaccharide (LPS). The p values of the significant comparisons are as follows: ^a^0.009 group S vs group P; ^b^0.048 group S vs group OP; ^c^0.012 group S vs group OP; ^d^0.034 vs the respective value at 0 hours. (**B**) Correlation between production of TNFα from PBMCs isolated at 48 hours and staphylococcal bone load before induction of pyelonephritis; the Spearman’s coefficient of correlation and the p-value of significance are provided.

The investigation of the mechanism of the protection of preceding osteomyelitis in the animals of OP group from death compared to the animals of P group comprised: (a) the innate immune response as expressed by the cytokine production of peripheral blood mononuclear cells (PBMCs); and (b) the potential of neutrophils for phagocytosis as expressed by tissue activity of myeloperoxidase (MPO). The main modulation in the production of tumor necrosis factor-alpha (TNFα) by PBMCs following stimulation with lipopolysaccharide (LPS) was found after 24 and 48 hours but not after 72 hours. More precisely, TNFα production by PBMCs of groups S and P steadily increased from baseline (0 hours) remaining, however, lower in group P than in group S. TNFα production by PBMCs of group OP remained stable compared to baseline being significantly lower compared to the other groups (Fig. 2A). The production of TNFα after stimulation with phytohemagglutin (PHA) was not remarkable in any group (data not shown). A negative correlation was found between the count of *S. aureus* in cancellous bone of animals of group OP sampled before the induction of pyelonephritis and the production of TNFα by PBMCs isolated at 48 hours post the induction of pyelonephritis (Fig. 2B). Interleukin (IL)-10 in PBMCs supernatants and serum ferritin levels at all time points were below the limit of detection. These findings point towards an attenuation of pro-inflammatory responses by PBMCs as part of the mechanism of protection from death in the OP group mainly expressed after 48 hours. This is compatible with the survival curves of the groups shown in Fig. 1 that start to separate after the first 48 hours.

**Figure 3:**
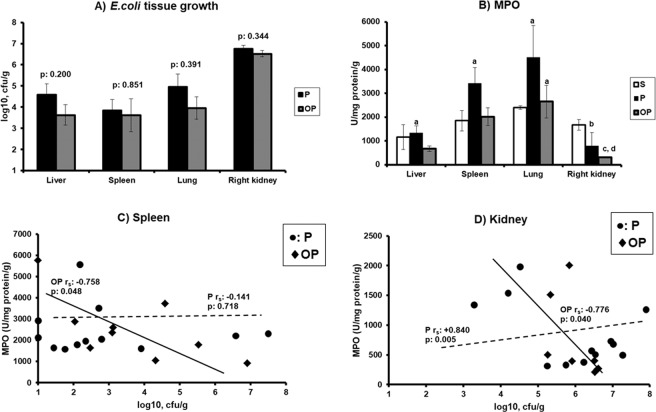
Modulation of the phagocytic potential in tissues Rabbits were divided into three groups: S subject to sham osteomyelitis followed three weeks later by sham pyelonephritis; P: subject to sham osteomyelitis followed three weeks later by *Escherichia coli* pyelonephritis; and OP: subject to *Staphylococcus aureus* osteomyelitis followed three weeks later by *E. coli* pyelonephritis. (**A**) Tissue outgrowth of *Escherichia coli*; p vales of comparisons between the P and the OP groups are provided. In all animals of the S group outgrowth was below the lower limit of detection. (**B**) Tissue concentrations of myeloperoxidase (MPO); The p values of the significant comparisons are provided: ^a^0.018 vs the respective values of the right kidney; ^b^0.002 group S vs group P; ^c^ < 0.0001 group S vs group OP; ^d^0.015 group P vs group OP. (**C**) Correlation between bacterial outgrowth and MPO in the spleen; the Spearman’s coefficient of correlation and the p-value of significance are provided for each group. D) Correlation between bacterial outgrowth and MPO in the right kidney; the Spearman’s coefficients of correlation and the p-values of significance are provided for each group.

The mean ± SE log10 cfu/g of *S. aureus* load in cancellous bone samples of group OP was 3.32 **±** 0.52 cfu/g. However, *S. aureus* did not grow in any other body tissue. The tissue bacterial load of *E. coli* was similar in groups P and OP (Fig. 3A). However, the tissue MPO activity that is a reflection of the level of infiltration by neutrophils^7^ was significantly attenuated in group OP compared to the other groups. As a matter of fact, the MPO activity of the spleen and lung of group P was remarkably increased compared to the MPO activity of the kidney of the same group where the primary infectious site was. This is compatible with a compartmentalization of neutrophils towards the remote organs where *E. coli* sepsis disseminated in group P; this did not happen with a similar intensity in group OP (Fig. 3B). As shown in Fig. 3C,D, there is negative correlation between *E. coli* load of the spleen and of the right kidney and of tissue MPO of the animals of the OP group. Although this may be an indicator of more efficient phagocytosis by neutrophils, other explanations may do apply.

**Figure 4:**
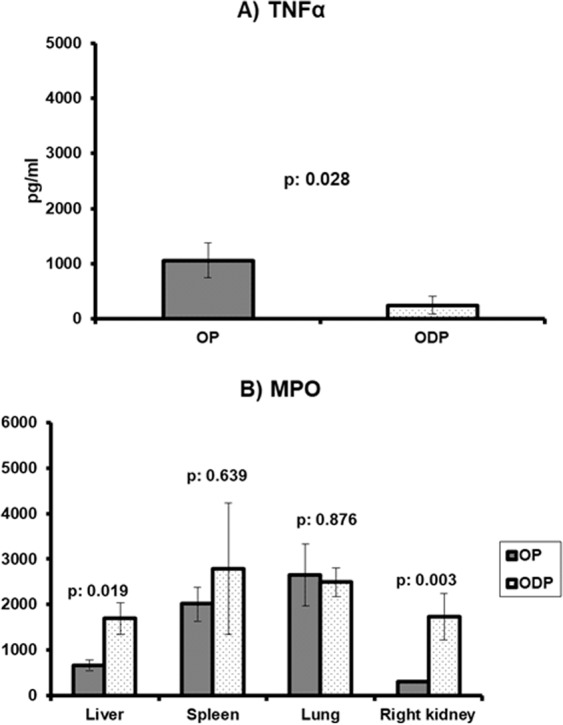
Effect of dalbavancin treatment Rabbits of the OP group were subject to *Staphylococcus aureus* osteomyelitis followed three weeks later by *E. coli* pyelonephritis; rabbits of the ODP group were subject to *Staphylococcus aureus* osteomyelitis, three weeks later received one single intravenous infusion of dalbavancin and one week later were subject to *E. coli* pyelonephritis (**A**) TNFα responses to bacterial lipopolysaccharide (LPS) of isolated peripheral blood mononuclear cells; and (**B**) comparative tissue myeloperoxidase activity. The p values of differences are provided.

The next question was how the production of TNFα and the tissue MPO activity might be modulated in case of rabbits receiving antistaphylococcal treatment. To this end, seven more rabbits were studied to which one single intravenous infusion of dalbavancin was administered at the time of removal of tibial foreign body three weeks from *S. aureus* challenge. Dalbavancin is a novel long-acting lipopeptide with high activity against *S. aureus* with resistance to methicillin as it was the studied pathogen. Rabbits were subject to *E. coli* pyelonephritis seven days after the infusion of dalbavancin (group ODP). In these rabbits, production of TNFα by PBMCs was even lower than the OP group (Fig. 4A). MPO activity in the liver and in the right kidney was greater than the OP group (Fig. 4B).

**Figure 5:**
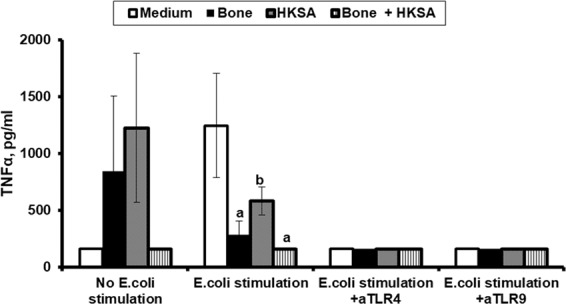
Modulation of TNFα production by pre-exposure to bone tissue and *Staphylococcus aureus*. Peripheral blood mononuclear cells of healthy rabbits were left unstimulated or were stimulated with heat-killed *Εscherichia coli* in the absence or presence of anti-TLR4 and anti-TLR9. Before stimulation, cells were pre-treated for 24 hours with medium, bone homogenate, heat-killed *Staphylococcus aureus* (HKSA) or their combination. Statistically significant comparisons are shown: ^a^p: 0.010 versus medium; ^b^p: 0.005 versus medium.

In order to better decipher the mechanism of TNFα modulation, the *in vivo* experiment was simulated *in vitro*. More precisely, rabbit PBMCs were pre-treated with bone homogenate, heat-killed *S. aureus* (HKSA) or both and then stimulated with heat-killed *E. coli* (HKEC). TNFα production was significantly decreased when cells were pre-treated with bone homogenate, HKSA or both (Fig. 5). This production was further decreased in the presence of anti-TLR (Toll-like receptor) 4 and anti-TLR9.

## Discussion

The present study showed that preceding *S. aureus* osteomyelitis offered survival benefit from *E. coli* pyelonephritis. In the studied model of sepsis, death is mediated through multiple organ dysfunctions as reflected by the intense infiltration of remote organs i.e. liver, spleen and lung by neutrophils where the pathogen was spread. Protection from death of rabbits with osteomyelitis was associated with attenuation of pro-inflammatory responses of *E. coli* sepsis as reflected by decreased production of TNFα by circulating PBMCs and by decrease of neutrophil inflammation in both the infected kidney and remote organs.

The mechanism behind this protection of the host resembles that of LPS tolerance. LPS tolerance is described as reduced immune responsiveness of immune cells to one LPS challenge subsequent to a previous exposure to *E. coli* or LPS^8,9^. However, a recent study using mammary epithelial cells from cows showed a similar phenomenon using agonists of TLR2 and TLR6. More precisely, cells were pre-treated with the TLR2/6 agonist Pam2CSK4 and subsequently exposed to LPS; these cells produced lower concentrations of TNFα than not pre-treated cells^10^. This phenomenon could not be reversed with the use of epigenetic inhibitors showing that epigenetic modifications do not mediate tolerance from TLR2/6 agonists. Since structures of *S. aureus* like muramyl dipeptide and peptidoglycan are mostly recognized via TLR2 interactions, this type of tolerance may be the mechanism underling the decreased TNFα production from the circulating PBMCs of the OP group. Interestingly, in our model, this type of tolerance needed a second *in vivo* hit to be shown since TNFα produced following *ex vivo* stimulation of PBMCs before the induction of pyelonephritis was not different between the OP and the P groups. From the clinical perspective, it needs to be outscored that the attenuation of TNFα responses was even more pronounced following antistaphylococcal treatment for one week. Probably in rabbits receiving dalbavancin treatment attenuated responses may be achieved not only through replicating *S. aureus* but also through dead staphylococcal remnants.

This phenomenon of TLR tolerance is fully dependent on the virulence of the pathogen. In a recent study, mice free from specific pathogens (SPF) were compared to mice cohoused with animal from pet stores (CoH). When exposed to low virulence *Listeria monocytogenes* that interacts with TLR2, CoH showed enhanced immune responses allowing better survival than SPF mice. The opposite took place when these mice were exposed to peritoneal sepsis mediated through cytokine storm^11^. In other terms, tolerance through TLR2 interaction is mostly favored in a setting of chronic well-tolerated infection. This type of infection was osteomyelitis in our setting.

However, the pathogenesis of bone infections is more complex since in parallel to the release of PAMPs, the immune system of the host is exposed to danger-associated molecular patterns (DAMPs) that are cellular constituents released by bone destruction. Examples of DAMPs are histones, adenosine triphosphate, urate and high mobility group box 1. DAMPs mostly interact with TLR4 and TLR9^12,13^. In our *in vitro* setting, DAMPs are contained in bone homogenates used for the stimulation of PBMCs. The blunt TNFα responses in the presence of anti-TLR4 and anti-TLR9 indicate that the attenuated TNFα production from the rabbits of the OP group is at least partly mediated through blockade of TLR4 and TLR9 on monocytes.

Sepsis following acute pyelonephritis by *E. coli* may rapidly deteriorate due to the development of sepsis-induced immunosuppression^14^. It may be hypothesized that tolerance developing after staphylococcal osteomyelitis prevented the development of immunosuppression.

The presented study showed for the first time that staphylococcal osteomyelitis modulated the innate immune responses of the host to such an extent that protected from death from a subsequent infection by highly virulent *E. coli*. The mechanism behind this phenomenon shares the characteristics of tolerance to TLR ligands and it is mediated through attenuated TNFα production and neutrophil activation. This phenomenon might probably be taken into account in light of the safety of several patients receiving outpatient antimicrobial therapy for chronic infections^15^.

## Animals and Methods

### Study design

This is an experimental study and no patients are involved. The experimental protocol received permission from the Ethics Committee of the Laboratory of Experimental Animals of ATTIKON University Hospital and the Veterinary Directorate of the Prefecture of Athens (N. 2578/29-05-2018), according to the Greek legislation in conformance with the 2010 Directive Council of the EU. A total of 46 male white New Zealand rabbits, aged 12 to 14 weeks with weight range between 3.0 and 3.5 kg were studied. Rabbits were housed in single metal cages, with access to tap water and standard balanced rabbit chow *ad libitum* throughout the study period. Temperature ranged between 18 °C and 22 °C, relative humidity was between 55% and 65%, and lighting was maintained in a 12 hours cycle. After one week of acclimatization, animals were randomly divided into three different groups, as follows:

- Sham surgery (S, n = 8) that was subject to tibia sham injury for 3 weeks followed by sham pyelonephritis
- Pyelonephritis group (P, n = 16) that was subject to tibia sham injury for 3 weeks followed by *E. coli* pyelonephritis
- Osteomyelitis and pyelonephritis group (OP, n = 12) that was subject to implant-related staphylococcal osteomyelitis of the tibia for 3 weeks followed by *E. coli* pyelonephritis.

Three more rabbits were used for blood sampling.

One isolate of *S. aureus* resistant to methicillin (MRSA) previously isolated from a patient with known implant-related osteomyelitis and another *E. coli* blood isolate from one previous patient with severe pyelonephritis were used for the induction of osteomyelitis and pyelonephritis respectively. Before each experiment, single colonies were suspended in Mueller-Hinton broth (Oxoid Ltd, London, UK) and incubated at 37 °C in a shaking water bath. The resulting inoculum was adjusted to 5 × 10^7^ cfu/ml and it was used for animal challenge.

On each day of experimentation at least one animal per group was operated using a randomization chart. Osteomyelitis was surgically induced in the right proximal tibial metaphysis in all rabbits of the OP group according to the guidelines provided by Mader^16^ and also described by our group^17,18^. Briefly, animals were sedated by the intramuscular injection of 25 mg/kg ketamine and 5 mg/kg xylazine (Alvet, Athens, Greece). Anaesthesia was maintained by the intramuscular administration of 15 mg/kg xylazine every 30 minutes. Following skin incision below the knee joint, exposing the medial cortex, a hole was made and one 18 G stainless steel needle was inserted to mechanically induce an endosteal trauma, followed by the instillation of 5 × 10^6^ colonies of MRSA at a volume of 0.1 ml. One SLS screw (3.2 mm × 10 mm, CliniLab, Athens, Greece) was used to seal the hole and serve as a foreign body. The skin over the insertion point was sutured with 3.0 silk suture and the animals were carried to their cages after recovery. The same procedure was followed for animals of groups S and P where 0.1 ml 0.9% sodium chloride was used for animal challenge instead of the MRSA pathogen. Rabbits then stayed in their cages being under closely monitoring and having access to food and water. Paracetamol suppositories were administered every 12 hours to minimize suffering.

After three weeks, the needle was removed and a small cancellous bone sample was obtained from the entry point for quantitative culture. Then animals were subject to obstructive *E. coli* pyelonephritis using the procedure already performed by our group^14,19–21^. Briefly, after midline abdominal incision and removal of the intestines, the right ureter was recognized and ligated just below the renal pelvis. One 0.3 ml suspension containing 1 × 10^7^ cfu/ml of the *E. coli* isolate was instilled into the renal pelvis, proximally to the ligation. After the end of the operation and for the first 48 hours rabbits were intramuscularly administered 1 mg/kg of meloxicam (Alvet, Athens, Greece) once daily. Analgesia with paracetamol suppositories 250 mg/kg was given to reduce suffering. Previous experiments of our group have shown that pain was well-managed through the use of paracetamol. This also allowed avoiding opiod sedation that modulates immune responses^22^.

Before the induction of pyelonephritis and at 24, 48 and 72 post pyelonephritis, six ml of heparinized blood was collected after venipuncture of the dorsal auricular artery. Survival was recorded every 12 hours for 14 days. Survivors were sacrificed by the intravenous administration of sodium thiopental (Alvet). After death or sacrifice, specimens of liver, spleen, lower lobe of the right lung and right kidney were aseptically collected into separate sterile containers with 0.9% N/S.

Some experiments were repeated with rabbits subject to osteomyelitis as described above (ODP group, n = 7). After three weeks, the needle was removed and rabbits were given one single intravenous infusion of 20 mg/kg of dalbavancin (Angelini, Athens, Greece), as done in previous experiments^23^. One week after the dalbavancin infusion, rabbits of this group were subject to pyelonephritis, as described above.

### Tissue handling

Tissue segments were weighed and mechanically homogenized using a grinder (Hilden, Germany). One aliquot of 0.1 ml was serially diluted 1:10 into Mueller-Hinton broth (Oxoid); 0.1 ml of each dilution was plated onto MacConkey agar (Oxoid). After incubation for 24 hours at 37 °C, the number of viable colonies was counted and expressed as log10 of colony forming units per gram tissue (cfu/g). The lower detection limit was 10 cfu/g. Removed needles were sonicated in a water bath for 3 min (BANDELIN BactoSonic, Berlin, Germany) and suspensions were quantitatively cultured onto Champan’s medium (Oxoid). Cancellous bone aliquots were also weighed and homogenized as described above and quantitatively cultured onto Chapman plates.

Activity of MPO was calculated as previously described^14^. Results were adjusted for tissue sample protein content on Bradford assay (Sigma-Aldrich) and they were expressed as MPO units/mg protein/g.

### Laboratory investigation

Peripheral blood mononuclear cells (PBMCs) were isolated after gradient centrifugation of heparinized whole blood over Ficoll-Hypaque (Biochrom AG, Berlin, Germany), washed and stimulated for cytokine production with purified 10 ng/ml LPS (lipopolysaccharide) of *E. coli* O55:B5 or 5 μg/ml PHA (InvivoGen, San Siego, USA), as previously described^14^. Concentrations of TNFα and IL-10 were measured in duplicate by an enzyme immunosorbent assay (R&D Inc, Minneapolis, USA). The lower detection limit was 156.5 pg/ml for TNFα; and 31.1 pg/ml for IL-10. Ferritin was measured by an enzyme immunoassay (R&D Inc) in all serum samples as a marker of acute pro-inflammation^24^; the lower detection limit was 75 ng/ml.

In separate experiments, cancellous bone was sampled from the tibia of three healthy rabbits as described above, weighted, homogenized and dissolved into 0.9% N/S. Before bone sampling, PMBCs were isolated from these rabbits and distributed at a density of 5 × 10^6^ cells/ml at the growth conditions mentioned above in duplicate. For these experiments, PBMCs were incubated in the absence or presence of 5 × 10^5^ cfu/ml of HKSA and/or 1 mg/ml of fresh bone homogenate. After 24 hours of incubation at 37 °C at 5% CO_2_, the medium was renewed and cells were left unstimulated or were stimulated with 5 × 10^5^ cfu/ml of HKEC. Experiments were repeated with 1 μg/ml of anti-TLR4 (LPS-EK, InvivoGen) or anti-TLR9 (ODN D-SL01, InvivoGen). After 24 hours of incubation, plates were centrifuged and TNFα was measured in supernatants by an enzyme immunosorbent assay.

### Power of the study and statistical analysis

The primary study endpoint was the comparative 14-day mortality between groups P and OP. Based on previous experiments^14,19–21^, the 14-day mortality of group P is anticipated to be 60%. With the assumption that this would have been decreased to 10% in the OP group, 14 rabbits per group would be needed to demonstrate this difference with 80% power at the 10% level of significance.

Survival was compared between groups by the log-rank test. Quantitative variables were expressed as mean ± SE. Comparisons between groups were done by ANOVA. Comparisons between serial time points within the same group were performed by the Wilcoxon ranked-sum test. Correlations between variables were performed using the Spearman’s rank of order. Any p value below 0.05 after correction for multiple comparisons was considered statistically significant.

#### List of ethics committees approving the study

The protocol was approved by the Ethics Committee of the Laboratory of Experimental Animals of ATTIKON University Hospital and the Veterinary Directorate of the Prefecture of Athens (No. 2578/29-05-2018).

## References

[1] Maffulli N (2016). The management of osteomyelitis in the adult. Surgeon.

[2] Metsemakers WJ (2018). Infection after fracture fixation: current surgical and microbiological concepts. Injury.

[3] Hung DZ (2017). Increased risk of chronic osteomyelitis after hip replacement: a retrospective population-based cohort study in an Asian population. Eur. J. Clin. Microbiol. Infect. Dis..

[4] Ahmed AI, Soliman RA, Samir S (2016). Cell free DNA and procalcitonin as early markers of complications in ICU patients with multiple trauma and major surgery. Clin. Lab..

[5] Chou SE (2019). Risk factors and complications contributing to mortality in elderly patients with fall-induced femoral fracture: A cross-sectional analysis based on trauma registry data of 2,407 patients. Int. J. Surg..

[6] Chousterman BG, Swirski FK, Weber GF (2017). Cytokine storm and sepsis disease pathogenesis. Semin. Immunopathol..

[7] Dai R (2017). Neutrophils and neutrophil serine proteases are increased in the spleens of estrogen-treated C57BL/6 mice and several strains of spontaneous lupus-prone mice. PLoS One.

[8] Cavaillon JM, Adib-Conquy M (2006). Bench-to-bedside review: endotoxin tolerance as a model of leukocyte reprogramming in sepsis. Crit. Care.

[9] Kopanakis K (2013). Pre-treatment with low dose endotoxin prolongs survival from experimental lethal endotoxic shock: benefit for lethal peritonitis by *Escherichia coli*. Cytokine.

[10] Günther J, Petzl W, Zerbe H, Schuberth HJ, Seyfert HM (2017). TLR ligands, but not modulators of histone modifiers, can induce the complex immune response pattern of endotoxin tolerance in mammary epithelial cells. Innate Immun..

[11] Huggings MA (2019). Microbial exposure enhances immunity to pathogens recognized by TLR2 but increase susceptibility to cytokine storm through TLR4 sensitization. Cell Rep..

[12] Zhou H (2019). Activation of both TLR and NOD signaling confers host innate immunity-mediated protection against microbial infection. Front. Immunol..

[13] Silk E, Zhao H, Weng H, Ma D (2017). The role of extracellular histones in organ injury. Cell Death Dis..

[14] Katsaris MP (2014). Immunomodulatory intervention with interferon-γ in *Escherichia coli* pyelonephritis. J. Urol..

[15] Allison GM (2014). Prediction model for 30-day hospital readmissions among patients discharged receiving outpatient parenteral antibiotic therapy. Clin. Infect. Dis..

[16] Mader JT (1985). Animal models of osteomyelitis. Am. J. Med..

[17] Kanellakopoulou K (2009). Treatment of experimental osteomyelitis by methicillin-resistant *Staphylococcus aureus* with a synthetic carrier of calcium sulphate (Stimulan^®^) releasing moxifloxacin. Int. J. Antimicrob. Agents.

[18] Kanellakopoulou K (2008). Local treatment of experimental *Pseudomonas aeruginosa* osteomyelitis with a biodegradable dilactide polymer releasing ciprofloxacin. Antimicrob. Agents Chemother..

[19] Giamarellos-Bourboulis EJ (2004). Immunomodulatory clarithromycin treatment of experimental sepsis and acute pyelonephritis caused by multidrug-resistant *Pseudomonas aeruginosa*. Antimicrob. Agents Chemother..

[20] Giamarellos-Bourboulis EJ (2005). Clarithromycin co-administered with amikacin attenuates systemic inflammation in experimental sepsis by *Escherichia coli*. Int. J. Antimicrob. Agents.

[21] Dimopoulos G (2015). Esmolol: immunomodulator in pyelonephritis by *Pseudomonas aeruginosa*. J. Surg. Res..

[22] Plein LM, Rittner HL (2018). Opioids and the immune system - friend or foe. Br. J. Pharmacol..

[23] Kussmann M (2018). Dalbavancin for treatment of implant-related methicillin-resistant *Staphylococcus aureus* osteomyelitis in an experimental rat model. Sci. Rep..

[24] Kyriazopoulou E (2017). Macrophage activation-like syndrome: an immunological entity associated with rapid progression to death in sepsis. BMC Med..

